# Significance of Serum Tumor Markers in Esophageal and Gastric Cancers: A Systematic Literature Review

**DOI:** 10.1002/ags3.70095

**Published:** 2025-09-15

**Authors:** Yasunori Matsumoto, Takeshi Toyozumi, Hideaki Shimada

**Affiliations:** ^1^ Department of Frontier Surgery Chiba University Graduate School of Medicine Chiba Japan; ^2^ Department of Gastroenterological Surgery and Clinical Oncology Toho University Graduate School of Medicine Tokyo Japan

**Keywords:** diagnosis, esophageal squamous cell carcinoma, gastric cancer, prognosis, tumor markers

## Abstract

**Aim:**

Serum tumor markers are helpful for diagnosis, monitoring treatment outcomes, and prognosis. However, their clinical utility for esophageal squamous cell carcinoma (ESCC) and gastric cancer (GC) remains unclear. This study aimed to comprehensively evaluate recent studies on serum tumor markers in ESCC and GC.

**Methods:**

We conducted a systematic review and meta‐analysis of studies published between January 2010 and March 2025 using PubMed. Overall, 84 and 468 articles on ESCC and GC were extracted, respectively. The sensitivity, specificity, positive predictive value, and summary receiver operating characteristic curves were calculated and evaluated using a systematic review and meta‐analysis. Prognostic values were assessed using the hazard ratio based on univariate and multivariate analyses.

**Results:**

In ESCC, squamous cell carcinoma antigen (SCC‐Ag) exhibited the highest sensitivity (38.7%) and positive predictive value (85.2%); moreover, it was the only independent prognostic factor across multiple articles. Carcinoembryonic antigen (CEA) and cytokeratin 19 fragment had low sensitivity and predictive value, whereas serum p53 antibody demonstrated moderate prognostic relevance. In GC, CEA and CA125 had the highest sensitivity (37.2%) and positive predictive value (72.2%), respectively, and CA125 and CA72‐4 were associated with peritoneal dissemination. CEA and CA125 levels were independent prognostic markers of GC.

**Conclusion:**

SCC‐Ag is a key marker for ESCC diagnosis and prognosis. CEA and CA125 are valuable markers for GC prognosis, whereas CA125 and CA72‐4 help detect peritoneal dissemination. Further research integrating the molecular characteristics of each tumor marker is warranted.

## Graphical abstract

The utility of serum tumor markers in esophageal squamous cell carcinoma and gastric cancer were evaluated by systematic review and meta‐analysis. In addition, the position of each marker, strategies for improving their accuracy in the future, and new markers are also described.

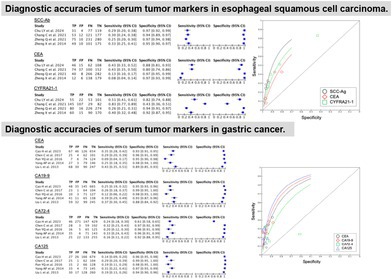

## 1 Introduction

Esophageal and gastric cancers are among the most prevalent gastrointestinal malignancies, characterized by high global morbidity and mortality, and rank among the leading causes of cancer‐related deaths. According to GLOBOCAN 2022 [1], roughly 970 000 gastric cancer cases are diagnosed annually, resulting in about 660 000 deaths, whereas esophageal cancer accounts for approximately 510 000 new cases and 450 000 deaths each year. The prognosis for esophageal and gastric cancers remains poor [2, 3], and their incidence rates are high in Asia, especially in China, Japan, and Korea. Dietary habits, living environment, infectious diseases such as *Helicobacter pylori* and Epstein–Barr virus, and genetic predisposition are believed to be intricately involved in these diseases [4].

Consequently, early diagnosis and straightforward monitoring of therapeutic response constitute critical clinical challenges to improving outcomes [5]. As a contribution to this, serum tumor markers that can be tested repeatedly with minimal invasiveness are considered inexpensive and useful in clinical practice [6, 7, 8].

Traditionally, serum tumor markers, such as carcinoembryonic antigen (CEA), squamous cell carcinoma antigen (SCC‐Ag), cytokeratin 19 fragment 21‐1 (CYFRA21‐1), and serum p53 antibody, have been widely used in the diagnosis of esophageal cancer [9, 10, 11]. Conversely, blood‐based tumor markers, such as CEA, carbohydrate antigen 19‐9 (CA19‐9), carbohydrate antigen 72‐4 (CA72‐4), and carbohydrate antigen 125 (CA125) have been widely used in gastric cancer [12, 13, 14]. Due to the limitations in sensitivity and specificity, the independent use of these markers is insufficient; therefore, efforts are underway to combine multiple markers and search for new ones [15, 16, 17]. Furthermore, most prior investigations of blood‐based tumor markers for esophageal cancer have combined esophageal adenocarcinoma with junctional cancer, and only a few studies have focused exclusively on esophageal squamous cell carcinoma (ESCC). Moreover, over the past decade, particularly in East Asia, there has been a significant increase in reports on blood‐based tumor markers for esophageal and gastric cancers [18, 19]; nonetheless, few systematic reviews have comprehensively analyzed these studies.

Therefore, this review aimed to comprehensively collate the latest evidence on serum tumor markers in ESCC and gastric cancer. Specifically, we conducted a systematic review of the standard serum markers reimbursed by public insurance in Japan and assessed their sensitivity, specificity, and prognostic value.

## 2 Methods

A search was conducted using PubMed from January 1, 2010, to March 31, 2025.

For ESCC, we searched the terms “esophageal cancer” in combination with various blood‐based tumor markers (search strategy and flow chart provided in the Figure S1 and Table S1) and identified 400 articles. Only studies on squamous cell carcinoma were considered; however, studies including other histologies, such as adenocarcinoma, were excluded. After omitting case reports and nonclinical studies, 84 articles were reviewed in full. The number of articles extracted was as follows: SCC‐Ag, 43; CEA, 42; CYFRA21‐1, 31; serum p53 antibody, 26; CA19‐9, 5; and a few on CA125, CA72‐4, and related markers. We conducted a meta‐analysis for four markers, SCC‐Ag, CEA, CYFRA21‐1, and serum p53 antibody, and undertook a systematic review of the clinical significance of each.

A search for “gastric cancer” and various tumor markers (see search formula and flow chart in the Figure S1 and Table S1) yielded 1108 articles. Case reports and nonclinical studies were excluded, and the abstracts or full texts of 468 articles were examined. The number of articles extracted was as follows: CEA, 368; CA19‐9, 115; CA72‐4, 115; CA125, 72; alpha‐fetoprotein (AFP), 77; carbohydrate antigen 242, 24; carbohydrate antigen 15‐3, 14; carbohydrate antigen 50, nine; CYFRA21‐1, six; and neuron‐specific enolase, three. Notably, AFP is often examined separately in studies on AFP‐producing gastric cancer and hepatoid adenocarcinoma of the stomach. Here, a meta‐analysis of four tumor markers, namely CEA, CA19‐9, CA72‐4, and CA125, along with a systematic review, was conducted to describe the clinical significance of each tumor marker.

Articles comparing cancer patients with healthy individuals or patients with benign diseases were included in the evaluation of diagnostic accuracy, and articles comparing cancer patients with and without metastasis were included in the evaluation of metastasis diagnosis. Articles evaluating sensitivity and prognosis in cancer patients were included regardless of the population.

A meta‐analysis was performed using RevMan Ver. 5.4 to calculate sensitivity, specificity, and summary receiver operating characteristics (SROC) curves. The quality of the included studies in the meta‐analysis was assessed using QUADAS‐2, and publication bias was assessed using a funnel plot. Hazard ratios (HRs) and 95% confidence intervals (CIs) were calculated in both univariate and multivariate analyses to examine prognoses.

## 3 Results

### 3.1 Sensitivity and Diagnostic Accuracy of Tumor Markers in ESCC

**FIGURE 1:**
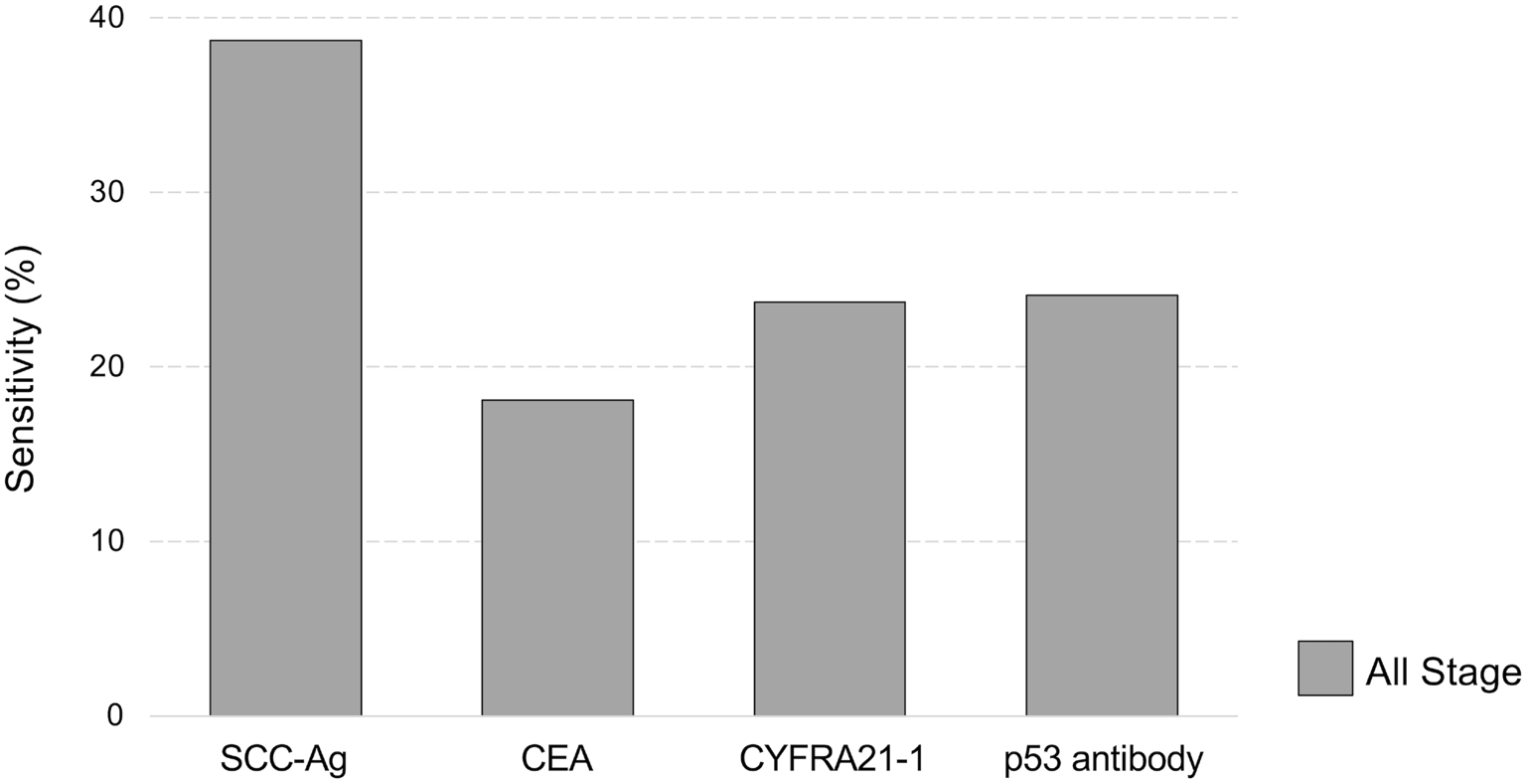
Sensitivity of serum tumor markers in all stages of ESCC. The overall sensitivity of each serum marker was 38.7%, 18.1%, 23.7%, and 24.1% for SCC‐Ag, CEA, CYFRA21‐1, and serum p53 antibody, respectively.

Few studies have simultaneously measured four blood‐based tumor markers for ESCC; therefore, we examined articles with a large number of cases analyzed for each marker [9, 20, 21, 22, 23, 24, 25, 26, 27, 28, 29, 30, 31, 32, 33, 34]. Table 1 presents the sensitivity, cutoff value, and patient stage distribution of the top five articles for each blood‐based tumor marker. Except for two articles [20, 25] that did not provide a clear explanation prior to treatment, the markers are pre‐treatment measurements. The cutoff values used in all articles are either consistent with previous reports or based on the manufacturer's recommended thresholds. The overall sensitivity for SCC‐Ag, CEA, CYFRA21‐1, and serum p53 antibodies was 38.7% (827/2136), 18.1% (276/1528), 23.7% (531/2239) CYFRA21‐1, and 24.1% (521/2164), respectively, when we simply add them together without considering their heterogeneity or bias (Figure 1).

**TABLE 1:** Sensitivity of serum tumor markers in ESCC.

Ref.	Author	Year	Number	Stage					SCC‐Ag	
				I	II	III	IV	N/A	Sensitivity	Cut off (ng/mL)
[21]	Suzuki T	2024	566	245	138	162	21	−	26.7%	1.5
[23]	Shinozuka T	2022	449	150	119	132	48	−	29.8%	1.5
[9]	Kanda M	2020	427	149	121	138	19	−	50.8%	1.1
[27]	Qiao Y	2019	315	147		168	−	−	15.6%	1.5
[33]	Cao X	2012	379	−	427	−	−	−	72.8%	1.5
Total			2136						38.7%	

Ref.	Author	Year	Number	Stage					CEA	
				I	II	III	IV	N/A	Sensitivity	Cut off (ng/mL)
[20]	Chen J	2025	173	52	59	28	14	20	73.4%	N/A
[23]	Shinozuka T	2022	449	150	119	132	48	−	9.4%	5.0
[25]	Zhang Q	2021	306	43	91	69	86	17	13.1%	5.0
[30]	Zhao H	2014	314	42	95	106	38	33	9.9%	20.0
[32]	Wang XB	2014	286	48	107	94	37	−	13.1%	5.0
Total			1528						18.1%	

				Stage					CYFRA21‐1	
Ref.	Author	Year	Number	I	II	III	IV	N/A	Sensitivity	cut off (ng/mL)
[24]	Ishioka N	2022	1047	224	258	456	109	−	9.9%	3.5
[25]	Zhang Q	2021	306	−	427	−	−	−	26.1%	3.3
[27]	Qiao Y	2019	315	147		168	−	−	26.3%	3.3
[33]	Cao X	2012	379	−	427	−	−	−	47.8%	3.3
[34]	Yan HJ	2012	192	—		—	—	192	43.2%	3.4
Total			2239						23.7%	

Ref.	Author	Year	Number	Stage					p53 antibody	
				I	II	III	IV	N/A	Sensitivity	Cut off (ng/mL)
[22]	Haneda R	2023	249	57	46	110	36	—	12.9%	1.3
[26]	Suzuki T	2021	1487	686	359	411	31	—	25.2%	1.3
[28]	Suzuki T	2018	135	47	28	55	5	—	21.5%	1.3
[29]	Yamashita K	2017	136	48		88		0	36.0%	0.5
[31]	Chai Y	2014	157	—	—	—	—	157	22.9%	N/A
Total			2164						24.1%	

*Note:* 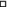, < 15%; 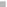, 15%–30%; 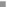, 30%–45%; 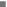, 45%–60%; 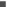, > 60%.

Abbreviation: N/A, not available.

**FIGURE 2:**
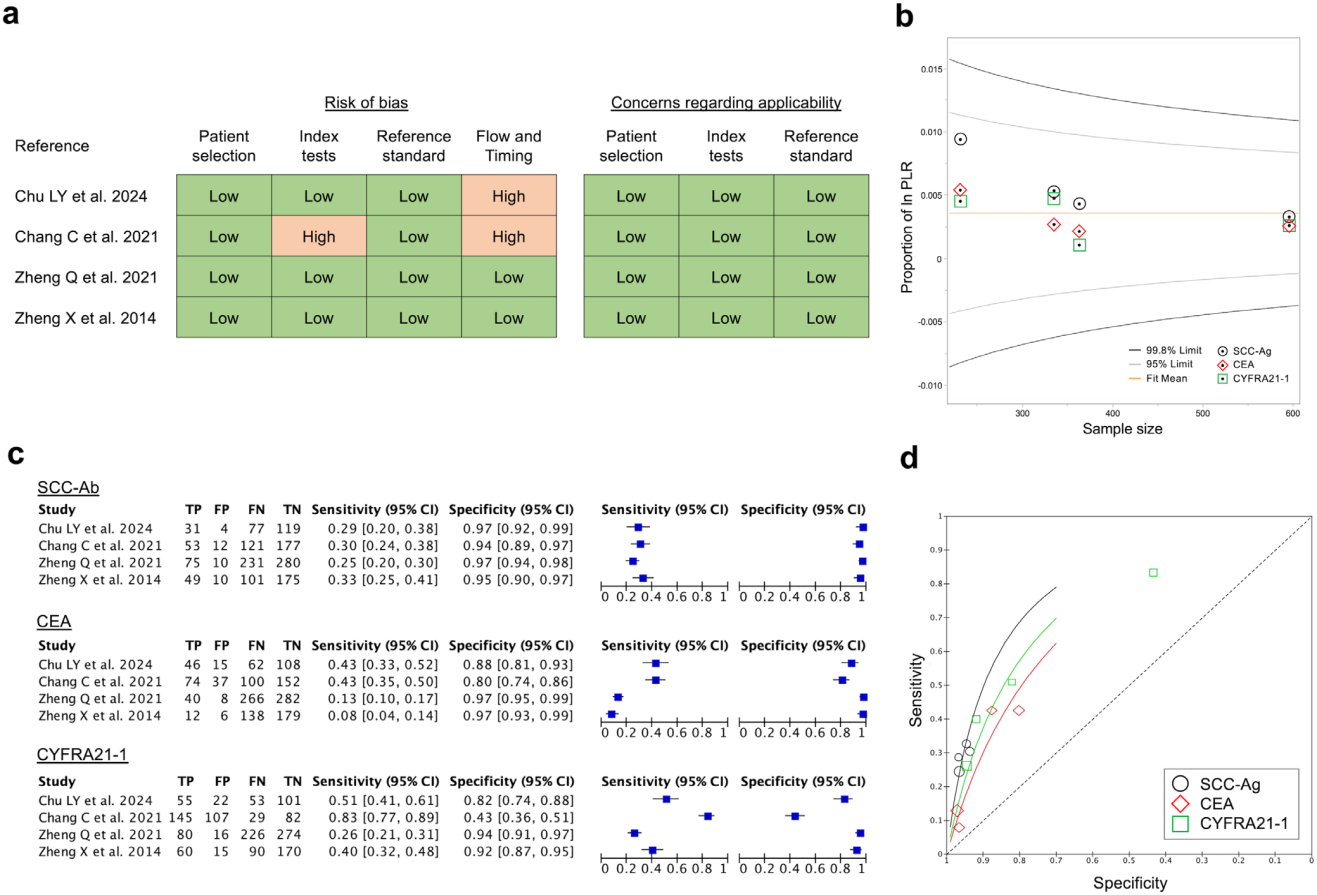
Diagnostic accuracies of serum tumor markers in ESCC. (a) Quality assessment of the four included articles using QUADAS‐2. (b) Funnel plots with sample size and positive likelihood ratios (PLR) were used to evaluate publication bias in the four articles. (c) The number of true positives (TN), false positives (FP), false negatives (FN), and true negatives (TN) was calculated from each of the four articles, and sensitivity and specificity were computed and plotted. (d) Summary ROC curve of each serum marker. SCC‐Ag had the highest AUC value, followed by CYFRA21‐1 and CEA.

Regarding diagnostic accuracy, the review question was set as “Which serum marker has the highest diagnostic ability for ESCC?” and we performed a meta‐analysis of four studies [25, 35, 36, 37] in which three markers, namely SCC‐Ag, CEA, and CYFRA21‐1, were measured concurrently, and true‐positive (TP), false‐positive (FP), and false‐negative (FN) rates were extracted. To date, no reports have simultaneously examined all four markers, including the p53 antibody, and computed TP, FP, and FN values. The quality of the literature was assessed using QUADAS‐2 (Figure 2a), and publication bias was assessed using a funnel plot based on the sample size and positive likelihood ratio in each article (Figure 2b). Details of the cases included in the articles (e.g., details of the control group, cutoff values used, etc.) are listed in the Table S2. The specificity of CYFRA21‐1was significantly below 0.7 in one article with own cutoff value; however, in the other studies, values were 0.8 or higher for all three markers. Moreover, sensitivity was 0.25–0.33, 0.08–0.43, and 0.26–0.83 for SCC‐Ag, CEA, and CYFRA21‐1, respectively, indicating that none of the markers was sufficiently sensitive (Figure 2c). The positive predictive values (PPVs) obtained by combining the four studies were 85.2%, 72.3%, and 68.0% for SCC‐Ag, CEA, and CYFRA21‐1, respectively. Although the PPV of SCC‐Ag was high, the PPVs of CYFRA21‐1 and CEA were approximately 70%, which cannot be considered high or meaningful. The SROC curves for each marker are illustrated in Figure 2d. Due to the limited number of articles included in this meta‐analysis, it is not possible to definitively state which method is superior in diagnostic ability. However, the area under the curve (AUC) values were highest for SCC‐Ag, followed by CYFRA21‐1 and CEA.

### 3.2 Prognostic Significance of Tumor Markers in ESCC

Overall, in 19 articles, univariate and multivariate analyses were conducted using overall survival as the endpoint and tumor markers as parameters. Table 2 presents articles with 200 or more cases analyzed [9, 21, 23, 24, 26, 27, 30, 33, 38, 39, 40], and in all articles, markers are pre‐treatment measurements. In univariate analysis, only one article reported an HR < 1.00 for CYFRA21‐1 (0.82). In other studies, positivity for all four markers was associated with an HR of 1.00 or higher, indicating a poor prognosis. Conversely, in multivariate analysis, with the exception of one report on CEA [33], the only tumor marker reported to be a significantly poor prognostic factor was SCC‐Ag; moreover, five articles [9, 23, 33, 38, 39] found that SCC‐Ag was an independent predictor of poor prognosis.

**TABLE 2:** Univariate and multivariate prognostic analysis of ESCC reviewed from the article.

Ref.	Author	Year	Number	Population	Univariate								Multivariate							
					SCC‐Ag		CEA		CYFRA21‐1		p53 antibody		SCC‐Ag		CEA		CYFRA21‐1		p53 antibody	
					HR	95% CI	HR	95% CI	HR	95% CI	HR	95% CI	HR	95% CI	HR	95% CI	HR	95% CI	HR	95% CI
[21]	Suzuki T	2024	566	UFS	N/A		N/A		N/A		N/A		1.05	0.73–1.53	N/A		N/A		N/A	
[23]	Shinozuka T	2022	449	UFS, NAC	2.2	1.44–3.35	N/A		1.12	0.54–2.32	N/A		1.82	1.19–2.79	N/A		—		N/A	
[24]	Ishioka N	2022	1047	UFS	N/A		N/A		N/A		N/A		N/A		1.22	0.70–2.14	N/A		N/A	
[38]	Kanie Y	2021	208	Recurrence	1.05	1.02–1.07	N/A		N/A		N/A		1.03	1.00–1.06	N/A		N/A		N/A	
[26]	Suzuki T	2021	1487	UFS	N/A		N/A		N/A		N/A		N/A		N/A		N/A		1.3	0.87–1.94
[39]	Okamura A	2021	453	NAC	N/A		N/A		N/A		N/A		1.79	1.25–2.56	N/A		N/A		N/A	
[9]	Kanda M	2020	427	UFS, NAC	2.26	1.51–3.38	N/A		0.82	0.40–1.69	N/A		2.04	1.36–3.07	N/A		—		N/A	
[27]	Qiao Y	2019	315	UFS	1.59	1.11–2.230	1.49	1.11–2.023	N/A		N/A		1.19	0.82–1.75	1.26	0.93–1.72	N/A		N/A	
[40]	Ma Q	2016	725	UFS	1.17	0.82–1.68	N/A		1.12	0.99–1.28	N/A		—		N/A		—		N/A	
[30]	Zhao H	2014	314	All treatment	N/A		N/A		1.08	0.530–2.23	N/A		N/A		—		N/A		N/A	
[33]	Cao X	2012	379	UFS (Stage II)	3.94	2.23–6.96	7.43	5.18–10.69	N/A		N/A		2.92	1.66–5.18	7.14	4.96–10.30	N/A		N/A	

*Note:* 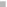, significant difference.

Abbreviations: CI, confidence interval; HR, hazard ratio; N/A, not available; NAC, neoadjuvant chemotherapy; UFS, upfront surgery.

### 3.3 Sensitivity and Diagnostic Accuracy of Tumor Markers in Gastric Cancer

**FIGURE 3:**
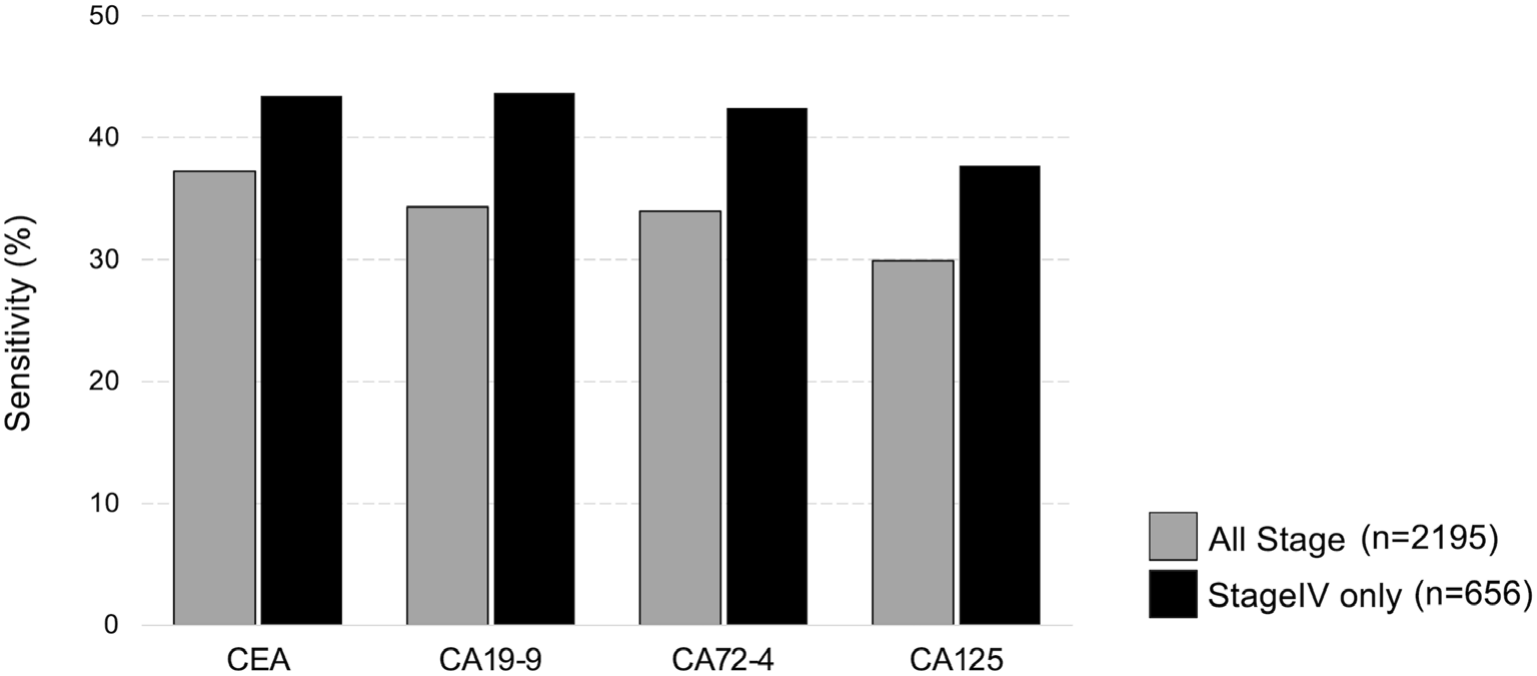
Sensitivity of serum tumor markers in all stages and stage IV gastric cancer. The sensitivities of each serum marker at all stages were 37.2%, 34.0%, 34.0%, and 29.9% for CEA, CA19‐9, CA72‐4, and CA125, respectively. The sensitivities of serum tumor markers in stage IV cases were 43.4%, 43.7%, 42.4%, and 37.7% for CEA, CA19‐9, CA72‐4, and CA125, respectively. The sensitivity of serum tumor markers in stage IV cases was higher than that in all other stages.

Table 3 presents the sensitivity, cutoff value, and patient stage distribution in 11 studies [17, 41, 42, 43, 44, 45, 46, 47, 48, 49, 50] that analyzed groups of 100–1000 patients with gastric cancer, simultaneously measuring four blood‐based tumor markers (CEA, CA19‐9, CA72‐4, and CA125). Except for two articles [17, 47] that did not provide a clear explanation prior to treatment, the markers are pre‐treatment measurements. The cutoff values used in all articles are either consistent with previous reports or based on the manufacturer's recommended thresholds. The sensitivities for each marker were 37.2% (773/2076), 34.3% (702/2045), 34.0% (665/1959), and 29.9% (564/1885) for CEA, CA19‐9, CA72‐4, and CA125, respectively, when we simply add them together without considering their heterogeneity or bias. The sensitivities in three articles [44, 47, 50] that only covered stage IV were 43.4% (285/656), 43.7% (272/623), 42.4% (249/587), and 37.7% (207/548) for CEA, CA19‐9, CA72‐4, and CA125, respectively. Compared with the sensitivity of 11 articles that included all stages, these rates were higher by approximately 6%–9% (Figure 3).

**TABLE 3:** Sensitivity of serum tumor markers in gastric cancer.

Ref.	Author	Year	Number	Stage					CEA		CA19‐9		CA72‐4		CA125	
				I	II	III	IV	N/A	Sensitivity	Cut off (ng/mL)	Sensitivity	Cut off (U/mL)	Sensitivity	Cut off (U/mL)	Sensitivity	Cut off (U/mL)
[41]	Zhang D	2024	215	88		123		4	57.2%	N/A	55.3%	N/A	65.6%	N/A	63.7%	N/A
[42]	Chen X	2024	128	58	28	42	0	—	10.2%	N/A	10.2%	N/A	13.3%	N/A	1.6%	N/A
[43]	Tong Y	2021	290	52	71	167	0	—	53.3%	1.6	25.2%	24.9	37.1%	4.6	27.7%	16
[44]	Abbas M	2019	216	0	0	0	216	—	58.8%	3.5	52.3%	39	30.1%	6.9	25.0%	35
[45]	Zhang K	2017	162	21	59	79	3	—	15.4%	5	22.8%	37	10.5%	10	25.9%	35
[46]	Sun Z	2014	184	53		126		5	34.8%	5	25.5%	37	29.9%	6.7	12.5%	20
[47]	Wang Q	2014	439	0	0	0	439	—	41.1%	10	40.0%	39	51.3%	6.9	45.9%	30.2
[17]	Yang AP	2014	106	—	—	—	—	106	25.5%	10	38.7%	37	33.0%	19.3	31.1%	35
[48]	Lai H	2014	215	—	—	—	—	215	13.5%	5	23.7%	37	21.4%	5	18.6%	35
[49]	Liu L	2013	138	22	28	63	25	—	44.9%	5	38.4%	39	15.9%	9.8	20.3%	40
[50]	Emoto S	2012	102	0	0	0	102	—	18.6%	5	36.3%	37	44.9%	4	46.1%	30
Total			2195						37.2%		34.3%		33.9%		29.9%	

*Note:* 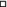, < 15%; 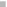, 15%–30%; 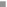, 30%–45%; 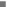, 45%–60%; 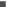, > 60%.

Abbreviation: N/A, not available.

**FIGURE 4:**
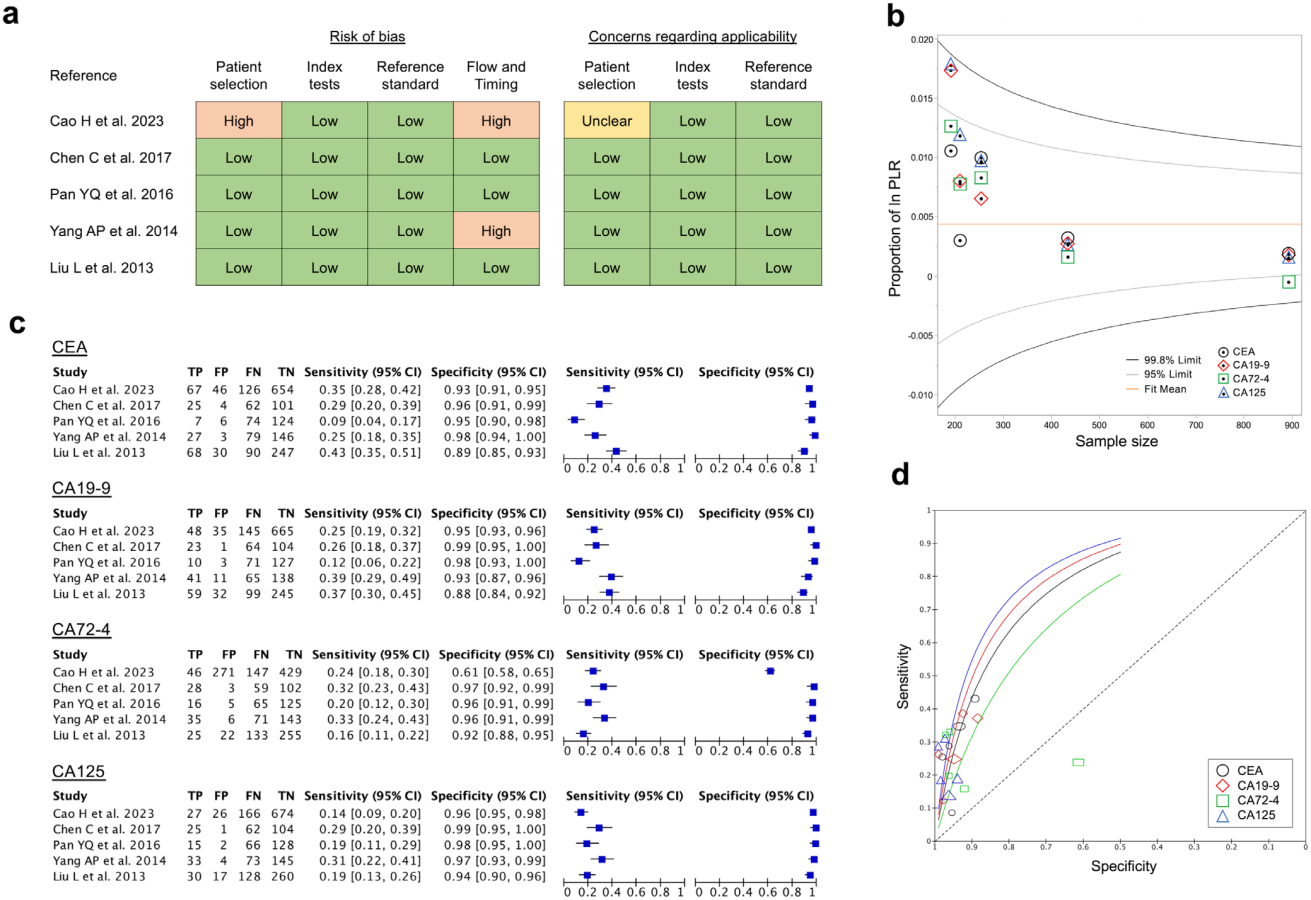
Diagnostic accuracies of serum tumor markers in gastric cancer. (a) Quality assessment of the five included articles using QUADAS‐2. (b) Funnel plots with sample size and positive likelihood ratios (PLR) were used to evaluate publication bias in the five articles. (c) The number of true positives (TP), false positives (FP), false negatives (FN), and true negatives (TN) was calculated from each of the five articles, and sensitivity and specificity were computed and plotted. (d) Summary ROC curve of each serum marker. The AUC value was highest for CA125, followed by CA19‐9, CEA, and CA72‐4.

Regarding diagnostic accuracy, eight studies [17, 45, 49, 50, 51, 52, 53, 54, 55] measured all four markers and reported AUCs of 0.55–0.83, 0.50–0.76, 0.54–0.84, and 0.50–0.73 for CEA, CA19‐9, CA72‐4, and CA125, respectively (Table S3). The review question was set as “Which serum marker has the highest diagnostic ability for GC?,” a meta‐analysis was performed on five articles [17, 49, 52, 55, 56] that simultaneously measured the TP, FP, FN, and FP of the four markers. The quality of the literature was assessed using QUADAS‐2 (Figure 4a), and publication bias was assessed using a funnel plot based on the sample size and positive likelihood ratio in each article (Figure 4b). Details of the cases included in the articles (e.g., details of the control group, cutoff values used, etc.) are listed in the Table S4. The specificity was below 0.7 for CA72‐4 in only one article [52] that include a large number of healthy controls and the possible presence of bias risk in QUADAS‐2; for CEA and CA19‐9 it was also 0.89 and 0.88 in one study, respectively, but in the other literature specificity for all four markers was 0.9 or higher. Conversely, the sensitivity was 0.09–0.43, 0.12–0.39, 0.16–0.33, and 0.14–0.31 for CEA, CA19‐9, CA72‐4, and CA125, respectively, suggesting that isolated markers do not possess sufficient sensitivity (Figure 4c). According to an integrated analysis of five articles, the PPVs for CEA, CA19‐9, CA72‐4, and CA125 were 68.6%, 68.8%, 48.9%, and 72.2%, indicating that CA125 was the most reliable marker of gastric cancer. The SROC curves for each marker are shown in Figure 4d. The AUC values were the highest for CA125, followed by CA19‐9, CEA, and CA72‐4.

### 3.4 Sensitivities of Metastases of Tumor Markers in Gastric Cancer

Only one article [48] reported the simultaneous measurement of four markers for lymph node metastasis, with reported sensitivities of 12.8%, 23.5%, 21.6%, and 15.7% for CA125 for CEA, CA19‐9, CA72‐4, and CA125, respectively. The sensitivity of CA19‐9 was relatively high; nonetheless, the sensitivity of each marker was less than 30%.

**FIGURE 5:**
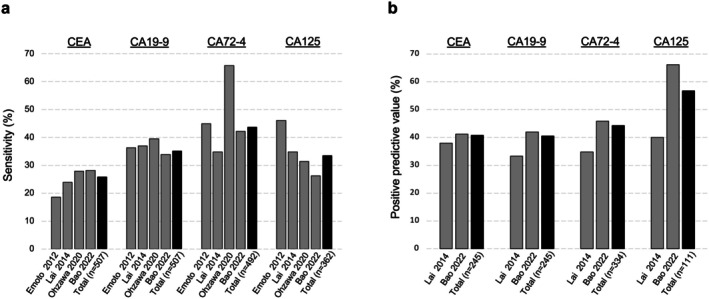
Sensitivity and positive predictive value of serum tumor markers in peritoneal dissemination of gastric cancer. (a) The sensitivity calculated by combining the reported articles was 25.8%, 35.1%, 43.7%, and 33.4% for CEA, CA19‐9, CA72‐4, and CA125, respectively. (b) The positive predictive value of CA125 was 56.8%, which was higher than that of the other three markers.

Four articles [48, 50, 57, 58] measured the four markers for peritoneal dissemination; the sensitivities of dissemination positivity, calculated from the number of cases listed, were 25.8%, 35.1%, 43.7%, and 33.4% for CEA, CA19‐9, CA72‐4, and CA125, respectively. The profile differed from the marker positivity rate in Stage IV cases mentioned previously. Specifically, the sensitivity of CEA was reported to be below 30% in all articles. However, the PPV of CA125 was 56.8%, which was higher than that of the other three markers (Figure 5).

### 3.5 Prognostic Significance of Tumor Markers in Gastric Cancer

Five articles [42, 43, 47, 50, 59] conducted univariate and multivariate analyses of the prognostic values of the four markers. Except one article [47] that did not provide a clear explanation prior to treatment, the markers are pre‐treatment measurements. In univariate analysis, one report of CA19‐9 found an HR of 0.80, while other reports showed an HR of 1 or more for positivity of all four markers. In multivariate analysis, high values of the three markers, except for CA19‐9, were significantly associated with poor prognosis (Table 4). In this analysis, CA125 had the highest HR in the univariate analysis of all articles, and CEA and CA125 were identified as independent poor prognostic factors in two articles in the multivariate analysis. However, the articles included in this analysis targeted different populations (e.g., the two studies that showed significant results for CA125 were metastatic or recurrent GC, while the other studies were surgical cases), and bias due to patient background and demographic differences must also be taken into consideration. Therefore, it is not feasible to conclude which marker more accurately reflects prognosis.

**TABLE 4:** Univariate and multivariate prognostic analysis of gastric cancer reviewed from the article.

Ref.	Author	Year	Number	Population	Univariate								Multivariate							
					CEA		CA19‐9		CA72‐4		CA125		CEA		CA19‐9		CA72‐4		CA125	
					HR	95% CI	HR	95% CI	HR	95% CI	HR	95% CI	HR	95% CI	HR	95% CI	HR	95% CI	HR	95% CI
[42]	Chen X	2024	128	UFS	2.75	1.01–7.48	0.80	0.19–3.42	1.93	0.64–5.76	4.17	0.97–19.01	−		−		−		−	
[43]	Tong Y	2021	290	NAC	1.19	0.84–2.07	1.73	1.19–2.52	2.03	1.39–2.97	2.34	1.52–3.61	1.27	0.71–2.25	0.98	0.53–1.83	2.18	1.17–4.06	1.50	0.82–2.75
[59]	Feng Y	2020	249	UFS	1.35	1.14–1.61	1.28	1.61–1.53	1.16	0.96–1.40	1.65	1.16–1.65	1.30	1.06–1.61	1.01	0.81–1.28	−		1.35	0.90–2.04
[47]	Wang Q	2014	439	MRGC	N/A		N/A		N/A		N/A		1.39	1.01–1.92	1.25	0.89–1.76	0.76	0.53–1.08	1.55	1.05–2.28
[50]	Emoto S	2012	102	PD	1.54	0.69–3.08	1.51	0.83–2.74	1.24	0.67–2.29	2.99	1.61–5.74	−		−		−		2.24	1.14–4.56

*Note:* 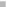, significant difference.

Abbreviations: CI, confidence interval; HR, hazard ratio; MRGC, metastatic or recurrent gastric cancer; N/A, not available; NAC, neoadjuvant chemotherapy; PD, peritoneal dissemination; UFS, upfront surgery.

## 4 Discussion

In this systematic review, we comprehensively evaluated the clinical and prognostic significance of SCC‐Ag, CEA, and CYFRA21‐1 in ESCC, as well as CEA, CA19‐9, CA72‐5, and CA125 in gastric cancer. SCC‐Ag exhibited the highest PPV for ESCC and was an independent poor prognostic factor for ESCC when prognosis was used as the endpoint. In gastric cancer, CEA exhibited the highest overall positivity; CA72‐4 demonstrated the greatest sensitivity for detecting peritoneal dissemination; and CA125 yielded the highest PPV. Moreover, CEA and CA125 concentrations have been identified as independent prognostic factors in gastric cancer.

### 4.1 SCC‐Ag in Esophageal SCC

SCC‐Ag was evaluated in most articles included in this systematic review. It displayed the highest reported sensitivity and can therefore be regarded as a useful tumor marker for ESCC. In addition, multivariate analyses based on overall survival yielded HRs of 1.03–2.92, underscoring its value as a prognostic parameter. Although each marker contributes to prognosis estimation to some extent, heterogeneity in patient background and selection bias preclude a definitive determination of which marker most accurately reflects prognosis.

### 4.2 CEA in ESCC

CEA was examined in the second‐largest number of articles after SCC‐Ag. Nonetheless, its pooled sensitivity in ESCC was only 18.1%, and the PPV derived from the meta‐analysis reached merely 72.3%. Consequently, the clinical relevance of CEA measurement in ESCC is limited.

### 4.3 CYFRA21‐1 in ESCC

The sensitivity of this analysis was slightly lower than that of the SCC‐Ag. However, when overall survival was used as the endpoint, CYFRA21‐1 was the second most frequently mentioned target in univariate analysis following SCC‐Ag, suggesting its usefulness as a prognostic factor for ESCC. Moreover, combining SCC‐Ag with CYFRA21‐1 has been proposed to improve prognostic assessment (HR = 1.371, 95% CI: 1.024–1.836, *p* = 0.034) [27], and integrating these markers with additional assays is expected to enhance diagnostic accuracy.

### 4.4 Serum p53 Antibody in ESCC

Investigations of serum p53 antibodies have been conducted predominantly in Japan. Their sensitivity is reportedly lower than that of SCC‐Ag but comparable to that of CYFRA21‐1. In the largest multi‐institutional cohort to date (*n* = 1487), serum p53 antibodies were identified as an independent prognostic factor [26]. Because few studies have assessed serum p53 concurrently with other markers, its relative utility in ESCC could not be fully evaluated here; additional research is therefore warranted.

### 4.5 CA19‐9 in ESCC

This marker was analyzed in only five articles. In a study that simultaneously analyzed SCC‐Ag, CEA, and CA19‐9, the sensitivity was reported to be extremely low at 4.9% [32]. Therefore, CA19‐9 is unlikely to be a useful diagnostic tool for ESCC.

### 4.6 CEA in Gastric Cancer

Throughout the search period, the status of this tumor marker remained unchanged, as it was the most frequently cited in the literature. Our analysis confirmed its high sensitivity in gastric cancer and its role as an independent prognostic factor. However, its sensitivity and PPV for detecting peritoneal dissemination remained low; thus, complementary markers are required.

### 4.7 CA19‐9 in Gastric Cancer

This marker did not demonstrate independence from other markers with respect to survival, and CEA and CA125, both associated with higher HRs, should therefore take precedence. For diagnosing peritoneal dissemination, it is inferior to CA72‐4 in sensitivity and PPV and to CA125 in PPV. Nonetheless, reported cutoff values vary only minimally, and the positive rate and sensitivity for peritoneal dissemination show the least variability, indicating that this marker can readily support clinical interpretation.

### 4.8 CA72‐4 in Gastric Cancer

Although the PPV of CA72‐4 for peritoneal dissemination is lower than that of CA125, it has the highest sensitivity and is therefore considered useful in detecting peritoneal dissemination. Cutoff values were inconsistent across the 11 articles reviewed; apart from two studies that employed 6.9 U/mL, each used a distinct threshold. A uniform standard is therefore needed to clarify its clinical value.

### 4.9 CA125 in Gastric Cancer

Due to its low sensitivity, CA125 detection has limited utility as a screening test for gastric cancer. However, it has the highest PPV for dissemination and is an independent prognostic factor, particularly in metastatic or recurrent GC; therefore, it may be useful for determining treatment options for advanced cancer.

### 4.10 Positioning of Serum Markers Now and in the Future

In Japanese guidelines [60, 61, 62], the recommended use of serum markers is limited. In esophageal squamous cell carcinoma, the Japanese guidelines do not clearly state the significance of measuring tumor markers. In gastric cancer, there is a weak recommendation to measure multiple markers and use them as a reference in determining the course of treatment for peritoneal dissemination. In addition, serum markers are considered to contribute to the early detection of recurrence and multiple cancers during postoperative follow‐up.

Based on the results of this review, the following markers should be considered in actual clinical practice, although not with sufficient accuracy, and further findings are expected in the future: SCC‐Ag is useful for screening ESCC, and its sensitivity may be enhanced by adding CYFRA21‐1 or serum p53 antibodies. For prognostication, SCC‐Ag alone or combined with CYFRA21‐1 is most informative. Moreover, CEA and CA19‐9 aid in screening for gastric cancer. Finally, CA72‐4 and CA125 are valuable for diagnosing peritoneal dissemination, whereas CEA and CA125 are informative for prognostic prediction.

In order to improve the accuracy of blood‐based tumor markers in the future, it will be important to improve accuracy through combinations of markers and to develop new markers. Regarding the diagnostic accuracy of GC, combining four markers yields a PPV of 84.1% [56], and AUC of 0.959 [17]. In ESCC, the combination of SCC‐Ag and CYFRA21‐1 has been reported to more sensitively reflect prognosis (HR: 1.371, 95% CI: 1.024–1.836, *p* = 0.034) [27], but large‐scale studies are lacking. Regarding new blood‐based markers such as circulating tumor DNA (ctDNA) and microRNA are expected to contribute to personalized medicine. ctDNA is already being used clinically for colorectal cancer, but in the field of ESCC and GC, it is still in the research stage [63, 64, 65], and evidence of its usefulness in actual clinical practice is eagerly awaited.

The limitations of this study are as follows. First, studies that included esophagogastric junction adenocarcinomas or esophageal adenocarcinomas were excluded when evaluating ESCC. Second, detailed clinicopathological variables, such as histological subtype, protein expression, gene profile, stage, and metastatic sites, remain unknown because a meta‐analysis using individual patient data has not yet been conducted. For instance, in stage IV gastric cancer versus peritoneal dissemination, Wang et al. [47] reported 439 cases of stage IV disease, with peritoneal, liver, and lung metastases observed in 64.0%, 36.8%, and 10.6% of patients, respectively, whereas Emoto et al. [50] analyzed 102 cases, all with peritoneal dissemination. Whether this disparity in disease composition accounts for the divergent marker profiles is unclear. Third, overlap among markers has not been verified in ESCC or gastric cancer, underscoring the need for multicenter studies and biobank‐based analyses. Fourth, no studies have examined therapeutic efficacy or chemotherapy responsiveness, and our review identified no detailed evaluations of immune checkpoint inhibitors. Fifth, there are no reports of cost‐effectiveness analyses or evaluations conducted during this review. In recent years, discussions have been held on new markers such as ctDNA, including cost‐effectiveness evaluations [66]. Serum markers can be measured at less than one‐hundredth of the cost of ctDNA and have the potential for high cost‐effectiveness, but this has not been confirmed.

In conclusion, our systematic review presents the current evidence of serum markers for ESCC and gastric cancer, presenting considerations for their use in medical practice. Future analyses should be conducted over time, as therapeutic efficacy and prognosis may change with the advent of new therapeutic agents.

## Author Contributions

**Yasunori Matsumoto:** conceptualization, methodology, software, data curation, investigation, validation, formal analysis, visualization, writing – original draft, writing – review and editing. **Takeshi Toyozumi:** conceptualization, methodology, data curation, investigation, validation, formal analysis, visualization, writing – review and editing. **Hideaki Shimada:** conceptualization, methodology, investigation, validation, supervision, visualization, project administration, writing – review and editing.

## Ethics Statement

The authors have nothing to report.

## Consent

The authors have nothing to report.

## Conflicts of Interest

The authors declare no conflicts of interest.

## Supporting information


**Figure S1:** Flowchart of the included articles for (a) esophageal squamous cell carcinoma and (b) gastric cancer.
**Table S1:** Search formulas used for PubMed database search.
**Table S2:** Case descriptions and cutoff values in the articles used for diagnostic accuracy analysis of esophageal squamous cell carcinoma.
**Table S3:** AUC values for diagnosis of gastric cancer by four tumor markers.
**Table S4:** Case descriptions and cutoff values in the articles used for diagnostic accuracy analysis of gastric cancer.

## Data Availability

The data that support the findings of this study are available from the corresponding author upon reasonable request.

## References

[1] F. Bray , M. Laversanne , H. Sung , et al., “Global Cancer Statistics 2022: GLOBOCAN Estimates of Incidence and Mortality Worldwide for 36 Cancers in 185 Countries,” CA: A Cancer Journal for Clinicians 74, no. 3 (2024): 229–263, 10.3322/caac.21834. 38572751

[2] D. Guo , J. Jin , D. Li , Y. He , and Y. Lin , “Analysis of the Incidence and Mortality Trends of Esophageal Cancer in Cancer Registry Areas of China and Japan,” International Journal of Cancer 155, no. 8 (2024): 1376–1386, 10.1002/ijc.35003. 38771567

[3] M. Asaka , M. Kobayashi , T. Kudo , et al., “Gastric Cancer Deaths by Age Group in Japan: Outlook on Preventive Measures for Elderly Adults,” Cancer Science 111, no. 10 (2020): 3845–3853, 10.1111/cas.14586. 32713120 PMC7540974

[4] H. Katoh and S. Ishikawa , “Lifestyles, Genetics, and Future Perspectives on Gastric Cancer in East Asian Populations,” Journal of Human Genetics 66, no. 9 (2021): 887–899, 10.1038/s10038-021-00960-8. 34267306 PMC8384627

[5] M. Sasako , “Progress in the Treatment of Gastric Cancer in Japan Over the Last 50 Years,” Annals of Gastroenterological Surgery 4, no. 1 (2020): 21–29, 10.1002/ags3.12306. 32021955 PMC6992673

[6] S. Holdenrieder , L. Pagliaro , D. Morgenstern , and F. Dayyani , “Clinically Meaningful Use of Blood Tumor Markers in Oncology,” BioMed Research International 2016 (2016): 9795269, 10.1155/2016/9795269. 28042579 PMC5155072

[7] H. Shimada , T. Noie , M. Ohashi , K. Oba , and Y. Takahashi , “Clinical Significance of Serum Tumor Markers for Gastric Cancer: A Systematic Review of Literature by the Task Force of the Japanese Gastric Cancer Association,” Gastric Cancer 17, no. 1 (2014): 26–33, 10.1007/s10120-013-0259-5. 23572188

[8] J. X. Jing , Y. Wang , X. Q. Xu , et al., “Tumor Markers for Diagnosis, Monitoring of Recurrence and Prognosis in Patients With Upper Gastrointestinal Tract Cancer,” Asian Pacific Journal of Cancer Prevention 15, no. 23 (2014): 10267–10272, 10.7314/apjcp.2014.15.23.10267. 25556459

[9] M. Kanda , M. Koike , D. Shimizu , et al., “Optimized Cutoff Value of Serum Squamous Cell Carcinoma Antigen Concentration Accurately Predicts Recurrence After Curative Resection of Squamous Cell Carcinoma of the Esophagus,” Annals of Surgical Oncology 27, no. 4 (2020): 1233–1240, 10.1245/s10434-019-07977-6. 31650302

[10] T. Nakamura , H. Ide , R. Eguchi , K. Hayashi , K. Takasaki , and S. Watanabe , “CYFRA 21‐1 as a Tumor Marker for Squamous Cell Carcinoma of the Esophagus,” Diseases of the Esophagus 11, no. 1 (2017): 35–39, 10.1093/dote/11.1.35. 29040480

[11] J. Zhang , Z. Xv , X. Wu , and K. Li , “Potential Diagnostic Value of Serum p53 Antibody for Detecting Esophageal Cancer: A Meta‐Analysis,” PLoS One 7, no. 12 (2012): e52896, 10.1371/journal.pone.0052896. 23285221 PMC3532438

[12] N. Wada , Y. Kurokawa , Y. Miyazaki , et al., “The Characteristics of the Serum Carcinoembryonic Antigen and Carbohydrate Antigen 19‐9 Levels in Gastric Cancer Cases,” Surgery Today 47, no. 2 (2017): 227–232, 10.1007/s00595-016-1408-3. 27566604

[13] T. Namikawa , Y. Kawanishi , K. Fujisawa , et al., “Serum Carbohydrate Antigen 125 Is a Significant Prognostic Marker in Patients With Unresectable Advanced or Recurrent Gastric Cancer,” Surgery Today 48, no. 4 (2018): 388–394, 10.1007/s00595-017-1598-3. 29043453

[14] Y. Xu , P. Zhang , K. Zhang , and C. Huang , “The Application of CA72‐4 in the Diagnosis, Prognosis, and Treatment of Gastric Cancer,” Biochimica Et Biophysica Acta. Reviews on Cancer 1876, no. 2 (2021): 188634, 10.1016/j.bbcan.2021.188634. 34656687

[15] S. Yajima , M. Ito , T. Suzuki , et al., “Application of Serum Anti‐ENO1 and Anti‐SSNA1 Antibody Biomarkers in Predicting the Prognosis of Gastric Cancer,” Oncology Letters 30, no. 1 (2025): 360, 10.3892/ol.2025.15106. 40469915 PMC12134978

[16] J. Kawada , T. Saito , Y. Kurokawa , et al., “Serum NY‐ESO‐1 and p53 Antibodies as Useful Tumor Markers in Gastric Cancer,” Annals of Gastroenterological Surgery 8, no. 2 (2023): 243–250, 10.1002/ags3.12757. 38455491 PMC10914697

[17] A. P. Yang , J. Liu , H. Y. Lei , Q. W. Zhang , L. Zhao , and G. H. Yang , “CA72‐4 Combined With CEA, CA125 and CAl9‐9 Improves the Sensitivity for the Early Diagnosis of Gastric Cancer,” Clinica Chimica Acta 437 (2014): 183–186, 10.1016/j.cca.2014.07.034. 25086284

[18] I. Hoshino , Y. Nabeya , N. Takiguchi , et al., “Inducing Multiple Antibodies to Treat Squamous Cell Esophageal Carcinoma,” BMC Cancer 20, no. 1 (2020): 1007, 10.1186/s12885-020-07466-0. 33069225 PMC7568359

[19] M. Kanda , Y. S. Suh , D. J. Park , et al., “Serum Levels of ANOS1 Serve as a Diagnostic Biomarker of Gastric Cancer: A Prospective Multicenter Observational Study,” Gastric Cancer 23, no. 2 (2020): 203–211, 10.1007/s10120-019-00995-z. 31377880

[20] J. Chen , Y. Zheng , Z. Wang , et al., “Development a Glycosylated Extracellular Vesicle‐Derived miRNA Signature for Early Detection of Esophageal Squamous Cell Carcinoma,” BMC Medicine 23, no. 1 (2025): 39, 10.1186/s12916-025-03871-z. 39849483 PMC11755925

[21] T. Suzuki , S. Yajima , A. Okamura , et al., “Prognostic Impact of Serum SCC Antigen in the 566 Upfront Surgery Group of Esophageal Squamous Cell Carcinoma: A Multi‐Institutional Study of the Japan Esophageal Society,” Annals of Thoracic and Cardiovascular Surgery 30, no. 1 (2024): 24–28, 10.5761/atcs.oa.24-00028. PMC11082496 38583987

[22] R. Haneda , S. Mayanagi , M. Inoue , et al., “Prognostic Impact of Perioperative Change in Serum p53 Antibody Titers in Esophageal Squamous Cell Carcinoma,” Esophagus 20, no. 4 (2023): 669–678, 10.1007/s10388-023-01013-z. 37212971

[23] T. Shinozuka , M. Kanda , D. Shimizu , et al., “Prognostic Value of a Modified Albumin‐Bilirubin Score Designed for Patients With Esophageal Squamous Cell Carcinoma After Radical Resection,” Annals of Surgical Oncology 29, no. 8 (2022): 4889–4896, 10.1245/s10434-022-11654-6. 35381933

[24] N. Ishioka , T. Suzuki , S. Yajima , et al., “Prognostic Impact of Pretreatment Serum CYFRA Status in 1047 Patients With Esophageal Squamous Cell Carcinoma Who Underwent Radical Resection: A Japan Esophageal Society Promotion Research,” Annals of Thoracic and Cardiovascular Surgery 28, no. 3 (2022): 163–170, 10.5761/atcs.oa.21-00195. 34690219 PMC9209890

[25] Q. Zheng , L. Zhang , M. Tu , et al., “Development of a Panel of Autoantibody Against NSG1 With CEA, CYFRA21‐1, and SCC‐Ag for the Diagnosis of Esophageal Squamous Cell Carcinoma,” Clinica Chimica Acta 520 (2021): 126–132, 10.1016/j.cca.2021.06.013. 34119530

[26] T. Suzuki , S. Yajima , A. Okamura , et al., “Clinical Impact of Preoperative Serum p53 Antibody Titers in 1487 Patients With Surgically Treated Esophageal Squamous Cell Carcinoma: A Multi‐Institutional Study,” Esophagus 18, no. 1 (2021): 65–71, 10.1007/s10388-020-00761-6. 32715348

[27] Y. Qiao , C. Chen , J. Yue , and Z. Yu , “Tumor Marker Index Based on Preoperative SCC and CYFRA 21‐1 Is a Significant Prognostic Factor for Patients With Resectable Esophageal Squamous Cell Carcinoma,” Cancer Biomarkers 25, no. 3 (2019): 243–250, 10.3233/CBM-190058. 31282406 PMC13082431

[28] T. Suzuki , S. Yajima , N. Ishioka , et al., “Prognostic Significance of High Serum p53 Antibody Titers in Patients With Esophageal Squamous Cell Carcinoma,” Esophagus 15, no. 4 (2018): 294–300, 10.1007/s10388-018-0629-5. 29959634

[29] K. Yamashita , T. Makino , K. Tanaka , et al., “Peritherapeutic Serum p53 Antibody Titers Are Predictors of Survival in Patients With Esophageal Squamous Cell Carcinoma Undergoing Neoadjuvant Chemotherapy and Surgery,” World Journal of Surgery 41, no. 6 (2017): 1566–1574, 10.1007/s00268-017-3894-x. 28108772

[30] H. Zhao , W. Chen , J. Wu , L. Wang , and W. Mao , “Clinical Significance of Preoperative Serum Tumor Markers in Esophageal Squamous Cell Carcinoma,” Journal of Cancer Research and Therapeutics 10, no. Suppl (2014): C179–C185, 10.4103/0973-1482.145863. 25450279

[31] Y. Chai , B. Peng , L. Dai , W. Qian , Y. Zhang , and J. Y. Zhang , “Autoantibodies Response to MDM2 and p53 in the Immunodiagnosis of Esophageal Squamous Cell Carcinoma,” Scandinavian Journal of Immunology 80, no. 5 (2014): 362–368, 10.1111/sji.12202. 24965442

[32] X. B. Wang , X. R. Jiang , X. Y. Yu , et al., “Macrophage Inhibitory Factor 1 Acts as a Potential Biomarker in Patients With Esophageal Squamous Cell Carcinoma and Is a Target for Antibody‐Based Therapy,” Cancer Science 105, no. 2 (2014): 176–185, 10.1111/cas.12331. 24383865 PMC4317821

[33] X. Cao , L. Zhang , G. R. Feng , et al., “Preoperative Cyfra21‐1 and SCC‐Ag Serum Titers Predict Survival in Patients With Stage II Esophageal Squamous Cell Carcinoma,” Journal of Translational Medicine 10 (2012): 197, 10.1186/1479-5876-10-197. 22999061 PMC3548759

[34] H. J. Yan , R. B. Wang , K. L. Zhu , et al., “Cytokeratin 19 Fragment Antigen 21‐1 as an Independent Predictor for Definitive Chemoradiotherapy Sensitivity in Esophageal Squamous Cell Carcinoma,” Chinese Medical Journal 125, no. 8 (2012): 1410–1415. 22613644

[35] L. Y. Chu , F. C. Wu , W. K. Fang , et al., “Secreted Proteins Encoded by Super Enhancer‐Driven Genes Could Be Promising Biomarkers for Early Detection of Esophageal Squamous Cell Carcinoma,” Biomedical Journal 47, no. 4 (2024): 100662, 10.1016/j.bj.2023.100662. 37774793 PMC11340493

[36] C. Chang , M. J. Wang , X. F. Bi , et al., “Elevated Serum Eotaxin and IP‐10 Levels as Potential Biomarkers for the Detection of Esophageal Squamous Cell Carcinoma,” Journal of Clinical Laboratory Analysis 35, no. 9 (2021): e23904, 10.1002/jcla.23904. 34288108 PMC8418505

[37] X. Zheng , S. Xing , X. M. Liu , et al., “Establishment of Using Serum YKL‐40 and SCCA in Combination for the Diagnosis of Patients With Esophageal Squamous Cell Carcinoma,” BMC Cancer 14 (2014): 490, 10.1186/1471-2407-14-490. 25001061 PMC4094903

[38] Y. Kanie , A. Okamura , S. Maruyama , et al., “Clinical Significance of Serum Squamous Cell Carcinoma Antigen for Patients With Recurrent Esophageal Squamous Cell Carcinoma,” Annals of Surgical Oncology 28, no. 12 (2021): 7990–7996, 10.1245/s10434-021-09945-5. 33839977

[39] A. Okamura , S. Matsuda , S. Mayanagi , et al., “Clinical Significance of Pretherapeutic Serum Squamous Cell Carcinoma Antigen Level in Patients With Neoadjuvant Chemotherapy for Esophageal Squamous Cell Carcinoma,” Annals of Surgical Oncology 28, no. 2 (2021): 1209–1216, 10.1245/s10434-020-08716-y. 32524457

[40] Q. Ma , W. Liu , R. Jia , et al., “Inflammation‐Based Prognostic System Predicts Postoperative Survival of Esophageal Carcinoma Patients With Normal Preoperative Serum Carcinoembryonic Antigen and Squamous Cell Carcinoma Antigen Levels,” World Journal of Surgical Oncology 14 (2016): 141, 10.1186/s12957-016-0878-5. 27151090 PMC4858859

[41] D. Zhang , R. H. Hu , X. M. Cui , X. H. Jiang , and S. Zhang , “Lipid Levels and Insulin Resistance Markers in Gastric Cancer Patients: Diagnostic and Prognostic Significance,” BMC Gastroenterology 24, no. 1 (2024): 373, 10.1186/s12876-024-03463-w. 39434031 PMC11495014

[42] X. Chen , Y. Zhang , Z. Liu , J. Song , and J. Li , “The Inflammation Score Predicts the Prognosis of Gastric Cancer Patients Undergoing Da Vinci Robot Surgery,” Journal of Robotic Surgery 18, no. 1 (2024): 131, 10.1007/s11701-024-01840-x. 38498240

[43] Y. Tong , Y. Zhao , Z. Shan , and J. Zhang , “CA724 Predicts Overall Survival in Locally Advanced Gastric Cancer Patients With Neoadjuvant Chemotherapy,” BMC Cancer 21, no. 1 (2021): 4, 10.1186/s12885-020-07666-8. 33402124 PMC7786973

[44] M. Abbas , A. Ahmed , G. J. Khan , et al., “Clinical Evaluation of Carcinoembryonic and Carbohydrate Antigens as Cancer Biomarkers to Monitor Palliative Chemotherapy in Advanced Stage Gastric Cancer,” Current Problems in Cancer 43, no. 1 (2019): 5–17, 10.1016/j.currproblcancer.2018.08.003. 30172422

[45] K. Zhang , H. Shi , H. Xi , et al., “Genome‐Wide lncRNA Microarray Profiling Identifies Novel Circulating lncRNAs for Detection of Gastric Cancer,” Theranostics 7, no. 1 (2017): 213–227, 10.7150/thno.16044. 28042329 PMC5196898

[46] Z. Sun and N. Zhang , “Clinical Evaluation of CEA, CA19‐9, CA72‐4 and CA125 in Gastric Cancer Patients With Neoadjuvant Chemotherapy,” World Journal of Surgical Oncology 12 (2014): 397, 10.1186/1477-7819-12-397. 25543664 PMC4320462

[47] Q. Wang , Y. Yang , Y. P. Zhang , et al., “Prognostic Value of Carbohydrate Tumor Markers and Inflammation‐Based Markers in Metastatic or Recurrent Gastric Cancer,” Medical Oncology 31, no. 12 (2014): 289, 10.1007/s12032-014-0289-9. 25344872

[48] H. Lai , Q. Jin , Y. Lin , et al., “Combined Use of Lysyl Oxidase, Carcino‐Embryonic Antigen, and Carbohydrate Antigens Improves the Sensitivity of Biomarkers in Predicting Lymph Node Metastasis and Peritoneal Metastasis in Gastric Cancer,” Tumour Biology 35, no. 10 (2014): 10547–10554, 10.1007/s13277-014-2355-5. 25060181 PMC4213369

[49] L. Liu , B. Yan , J. Huang , et al., “The Identification and Characterization of Novel N‐Glycan‐Based Biomarkers in Gastric Cancer,” PLoS One 8, no. 10 (2013): e77821, 10.1371/journal.pone.0077821. 24147084 PMC3798316

[50] S. Emoto , H. Ishigami , H. Yamashita , H. Yamaguchi , S. Kaisaki , and J. Kitayama , “Clinical Significance of CA125 and CA72‐4 in Gastric Cancer With Peritoneal Dissemination,” Gastric Cancer 15, no. 2 (2012): 154–161, 10.1007/s10120-011-0091-8. 21892754

[51] X. Li , Y. L. Lin , J. K. Shao , et al., “Plasma Exosomal hsa_circ_0079439 as a Novel Biomarker for Early Detection of Gastric Cancer,” World Journal of Gastroenterology 29, no. 22 (2023): 3482–3496, 10.3748/wjg.v29.i22.3482. 37389236 PMC10303519

[52] H. Cao , L. Zhu , L. Li , W. Wang , and X. Niu , “Serum CA724 Has No Diagnostic Value for Gastrointestinal Tumors,” Clinical and Experimental Medicine 23, no. 6 (2023): 2433–2442, 10.1007/s10238-023-01025-0. 36920593 PMC10543537

[53] H. Jiang , S. Guo , Y. Zhao , et al., “Circulating Long Non‐Coding RNA PCGEM1 as a Novel Biomarker for Gastric Cancer Diagnosis,” Pathology, Research and Practice 215, no. 10 (2019): 152569, 10.1016/j.prp.2019.152569. 31421977

[54] S. L. Wang , G. Y. Yu , J. Yao , Z. S. Li , A. R. Mao , and Y. Bai , “Diagnostic Role of Carbohydrate Antigen 72‐4 for Gastrointestinal Malignancy Screening in Chinese Patients: A Prospective Study,” Journal of Digestive Diseases 19, no. 11 (2018): 685–692, 10.1111/1751-2980.12681. 30345716

[55] Y. Q. Pan , Y. Y. Ruan , J. B. Peng , et al., “Diagnostic Significance of Soluble Human Leukocyte Antigen‐G for Gastric Cancer,” Human Immunology 77, no. 4 (2016): 317–324, 10.1016/j.humimm.2016.01.009. 26788811

[56] C. Chen , Q. Chen , Q. Zhao , M. Liu , and J. Guo , “Value of Combined Detection of Serum CEA, CA72‐4, CA19‐9, CA15‐3 and CA12‐5 in the Diagnosis of Gastric Cancer,” Annals of Clinical and Laboratory Science 47, no. 3 (2017): 260–263. 28667025

[57] D. Bao , Z. Yang , S. Chen , K. Li , and Y. Hu , “Construction of a Nomogram Model for Predicting Peritoneal Dissemination in Gastric Cancer Based on Clinicopathologic Features and Preoperative Serum Tumor Markers,” Frontiers in Oncology 12 (2022): 844786, 10.3389/fonc.2022.844786. 35719995 PMC9198602

[58] H. Ohzawa , Y. Kimura , A. Saito , et al., “Ratios of miRNAs in Peritoneal Exosomes Are Useful Biomarkers to Predict Tumor Response to Intraperitoneal Chemotherapy in Patients With Peritoneal Metastases From Gastric Cancer,” Annals of Surgical Oncology 27, no. 13 (2020): 5057–5064, 10.1245/s10434-020-09007-2. 32804324

[59] Y. Feng , Y. Jiang , Q. Zhao , J. Liu , H. Zhang , and Q. Chen , “Long‐Term Outcomes and Prognostic Factor Analysis of Resected Siewert Type II Adenocarcinoma of Esophagogastric Junction in China: A Seven‐Year Study,” BMC Surgery 20, no. 1 (2020): 302, 10.1186/s12893-020-00926-1. 33256690 PMC7706258

[60] Y. Kitagawa , R. Ishihara , H. Ishikawa , et al., “Esophageal Cancer Practice Guidelines 2022 Edited by the Japan Esophageal Society: Part 1,” Esophagus 20, no. 3 (2023): 343–372, 10.1007/s10388-023-00993-2. 36933136 PMC10024303

[61] Japanese Gastric Cancer Association , “Japanese Gastric Cancer Treatment Guidelines 2021 (6th Edition),” Gastric Cancer 26, no. 1 (2023): 1–25, 10.1007/s10120-022-01331-8. 36342574 PMC9813208

[62] K. Matsusaki , K. Aridome , S. Emoto , et al., “Clinical Practice Guideline for the Treatment of Malignant Ascites: Section Summary in Clinical Practice Guideline for Peritoneal Dissemination (2021),” International Journal of Clinical Oncology 27, no. 1 (2022): 1–6, 10.1007/s10147-021-02077-6. 34800177 PMC8732893

[63] T. Iwaya , F. Endo , F. Takahashi , T. Tokino , Y. Sasaki , and S. S. Nishizuka , “Frequent Tumor Burden Monitoring of Esophageal Squamous Cell Carcinoma With Circulating Tumor DNA Using Individually Designed Digital Polymerase Chain Reaction,” Gastroenterology 160, no. 1 (2021): 463–465.e4, 10.1053/j.gastro.2020.09.035. 33011175

[64] J. Mencel , S. Slater , E. Cartwright , and N. Starling , “The Role of ctDNA in Gastric Cancer,” Cancers 14, no. 20 (2022): 5105, 10.3390/cancers14205105. 36291888 PMC9600786

[65] S. Nagano , Y. Kurokawa , T. Hagi , et al., “Extensive Methylation Analysis of Circulating Tumor DNA in Plasma of Patients With Gastric Cancer,” Scientific Reports 14, no. 1 (2024): 30739, 10.1038/s41598-024-79252-y. 39730450 PMC11680901

[66] Y. Li , A. K. Heer , H. S. Sloane , et al., “Budget Impact Analysis of Circulating Tumor DNA Testing for Colon Cancer in Commercial Health and Medicare Advantage Plans,” JAMA Health Forum 5, no. 5 (2024): e241270, 10.1001/jamahealthforum.2024.1270. 38819797 PMC11143467

