# Supplementary material for: Significance of Serum Tumor Markers in Esophageal and Gastric Cancers: A Systematic Literature Review
Source: Ann Gastroenterol Surg. 2025 Sep 15;10(1):54–66. doi: 10.1002/ags3.70095 (PMC12757164; doi:10.1002/ags3.70095)
Supplement: Supplementary file 1 — Figure S1: Flowchart of the included articles for (a) esophageal squamous cell carcinoma and (b) gastric cancer. Table S1: Search formulas used for PubMed database search. Table S2: Case descriptions and cutoff values in the articles used for diagnostic accuracy analysis of esophageal squamous cell carcinoma. Table S3: AUC values for diagnosis of gastric cancer by four tumor markers. Table S4: Case descriptions and cutoff values in the articles used for diagnostic accuracy analysis of gastric cancer. [file AGS3-10-54-s001.docx]

**Supplementary materials**


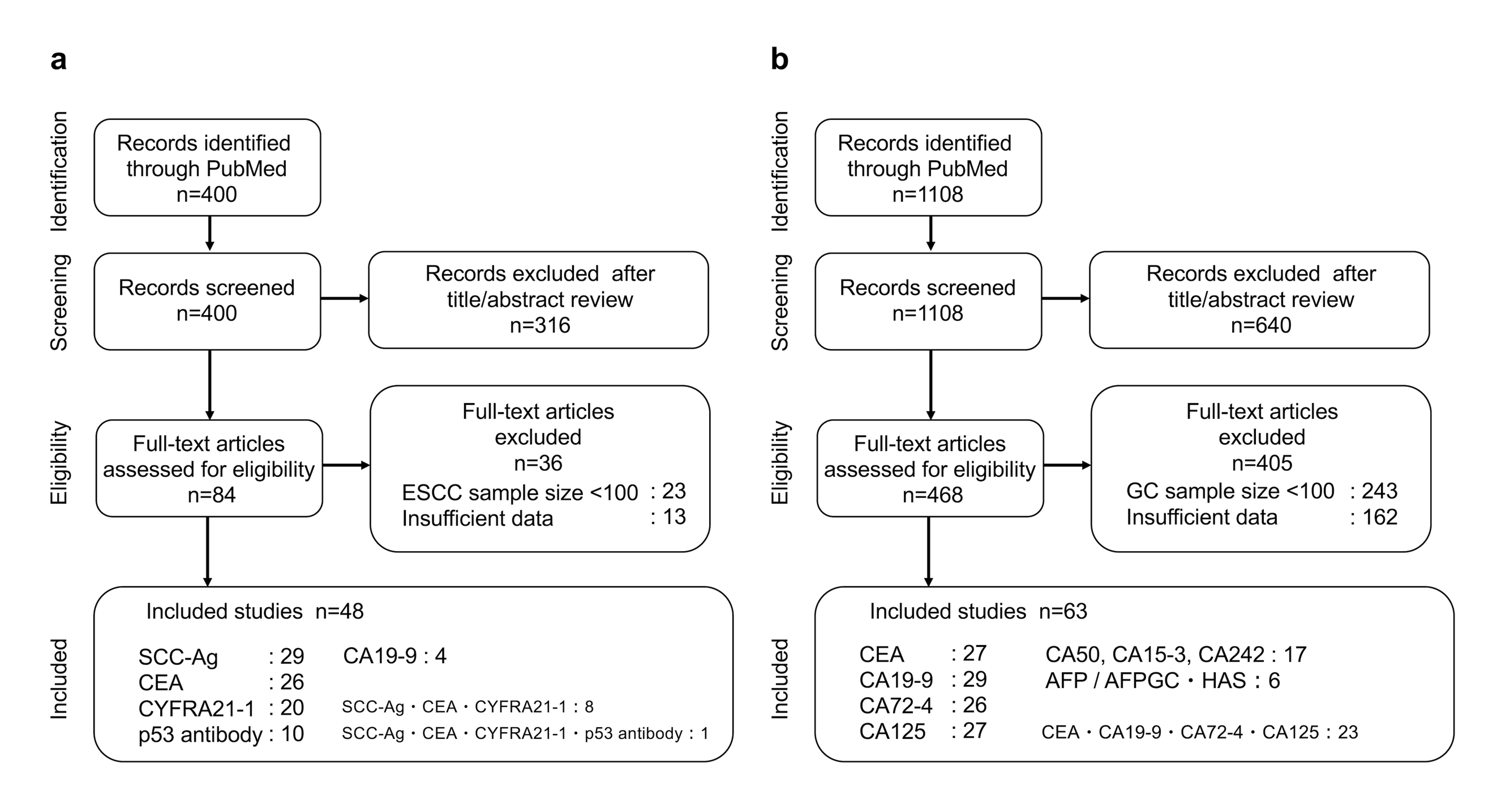


Supplementary Figure S1

Flowchart of the included articles for (a) esophageal squamous cell carcinoma and (b) gastric cancer. ESCC, esophageal squamous cell carcinoma; GC, gastric cancer; SCC-Ag, squamous cell carcinoma antigen; CEA, carcinoembryonic antigen; CYFRA, cytokeratin 19 fragment; CA, carbohydrate antigen; AFP, alpha-fetoprotein; AFPGC, alpha-fetoprotein-producing gastric cancer; HAS, hepatoid adenocarcinoma of the stomach.

Supplementary Table S1

Search formulas used for PubMed database search

| ESCC | (esophagus[TIAB] OR esophageal[TIAB] ) AND (cancer[ TIAB] OR carcinoma[TIAB] OR squamous cell carcinoma[TIAB]) AND (serum[TIAB] OR plasma[TIAB] OR blood[TIAB]) AND (marker[TIAB] OR SCC[TIAB] OR CEA[ TIAB] OR CYFRA[TIAB] OR p53[TIAB] OR TP53[TIAB] OR CA19-9[TIAB] OR CA50[TIAB] OR Cytokeratin[TIAB] OR Carcinoembryonic[TIAB]) AND 2010/01/01:2025/03/31[Date - Publication] AND "humans"[MeSH Terms] AND ("English"[Language] OR "Japanese"[Language]) | 400 articles |
| --- | --- | --- |
| GC | (stomach[TIAB] OR gastric[TIAB] OR esophagogastric[TIAB] ) AND (cancer[TIAB] OR carcinoma[TIAB] OR adenocarcinoma[TIAB]) AND (serum[TIAB] OR plasma[TIAB] OR blood[TIAB]) AND (marker[TIAB] OR CA19-9[TIAB] OR CEA[TIAB] OR AFP[TIAB] OR CA72-4[TIAB] OR CA125[TIAB] OR STN[TIAB] OR TPA[TIAB] OR IPA[TIAB] OR Carbohydrate[TIAB] OR Carcinoembryonic[TIAB]) AND 2010/01/01:2025/03/31[Date - Publication] AND "humans"[MeSH Terms] AND ("English"[Language] OR "Japanese"[Language]) | 1108 articles |
| ESCC, esophageal squamous cell carcinoma; GC, gastric cancer | |  |

Supplementary Table S2

Case descriptions and cutoff values in the articles used for diagnostic accuracy analysis of esophageal squamous cell carcinoma

| Ref. | Author | Year | Period | ESCC | Benign | HC |  | Cut off value | | |
| --- | --- | --- | --- | --- | --- | --- | --- | --- | --- | --- |
|  |  |  |  |  |  |  |  | SCC-Ag | CEA | CYFRA21-1 |
| [35] | Chu LY | 2024 | 2017 - 2018 | 108 | 0 | 123 |  | 2.7 ng/ml | 3.8 ng/ml | 3.3 ng/ml |
| [36] | Chang C | 2021 | N/A | 174 | 0 | 189 |  | 1.02μg/ml | 2.01ng/ml | 1.22ng/ml |
| [25] | Zheng Q | 2021 | 2016 - 2018 | 306 | 135 | 155 |  | 1.5 ng/ml | 5.0 ng/ml | 3.3 ng/ml |
| [37] | Zheng X | 2014 | 2002 - 2005 | 150 | 59 | 126 |  | 1.5 ng/ml | 5.0 ng/ml | 3.3 ng/ml |
| ESCC, esophageal squamous cell carcinoma; HC, healthy control | | | | | | | | | | |

Supplementary Table S3

AUC values for diagnosis of gastric cancer by four tumor markers

|  |  |  |  |  |  |  | AUC | | | |
| --- | --- | --- | --- | --- | --- | --- | --- | --- | --- | --- |
| Ref. | Author | Year | GC | cont | Benign / HC |  | CEA | CA19-9 | CA72-4 | CA125 |
| [51] | Li X | 2023 | 240 | 63 | 0 / 63 |  | 0.59 | 0.57 | 0.54 | 0.50 |
| [52] | Cao H | 2023 | 193 | 700 | 0 / 700 |  | 0.75 | 0.57 | 0.60 | 0.59 |
| [53] | Jiang H | 2019 | 317 | 100 | 0 / 100 |  | 0.66 | 0.57 | 0.62 | 0.66 |
| [54] | Wang SL | 2018 | 86 | 35 | 0 / 35 |  | 0.57 | 0.50 | 0.62 | 0.58 |
| [45] | Zhang K | 2017 | 30 | 30 | 0 / 30 |  | 0.55 | 0.51 | 0.66 | 0.69 |
| [45] | Zhang K | 2017 | 80 | 80 | 0 / 80 |  | 0.57 | 0.60 | 0.61 | 0.59 |
| [55] | Pan YQ | 2016 | 81 | 130 | 53 / 77 |  | 0.65 | 0.64 | 0.58 | 0.63 |
| [17] | Yang AP | 2014 | 106 | 149 | 149 / 0 |  | 0.83 | 0.72 | 0.84 | 0.73 |
| [49] | Liu L | 2013 | 158 | 277 | 129 / 148 |  | 0.74 | 0.76 | 0.67 | 0.72 |
| AUC, area under the curve; GC, gastric cancer; HC, healthy control | | | | | | | | | | |

Supplementary Table S4

Case descriptions and cutoff values in the articles used for diagnostic accuracy analysis of gastric cancer

| Ref. | Author | Year | Period | GC | Benign | HC |  | Cutoff value | | | |
| --- | --- | --- | --- | --- | --- | --- | --- | --- | --- | --- | --- |
|  |  |  |  |  |  |  |  | CEA | CA19-9 | CA72-4 | CA125 |
| [52] | Cao H | 2023 | 2020 - 2022 | 193 | 0 | 700 |  | 5 ng/ml | 37 U/ml | 7 U/ml | 35 U/ml |
| [56] | Chen C | 2017 | 2014 - 2015 | 87 | 65 | 40 |  | 5 ng/ml | 25 ng/ml | 8.2 ng/ml | 14 ng/ml |
| [55] | Pan YQ | 2016 | 2009 - 2014 | 81 | 53 | 77 |  | 5 ng/ml | 37 U/ml | 6.9 U/ml | 35 U/ml |
| [17] | Yang AP | 2014 | 2011 - 2013 | 106 | 149 | 0 |  | 10 ng/ml | 37 U/ml | 19.3 U/ml | 35 U/ml |
| [49] | Liu L | 2013 | 2010 - 2012 | 158 | 129 | 148 |  | 5 ng/ml | 39 U/ml | 9.8 U/ml | 40 U/ml |
| GC, gastric cancer; HC, healthy control | | | | | | | | | | | |
